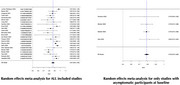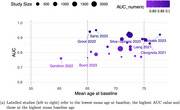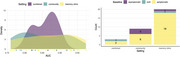# Longitudinal Studies of Blood‐Based Biomarkers for Dementia in Primary Care: A Systematic Review and Meta‐Analysis

**DOI:** 10.1002/alz70856_103017

**Published:** 2025-12-26

**Authors:** Rozelle C Kane, Leonidas Chouliaras, Simon Griffin

**Affiliations:** ^1^ University of Cambridge, CAMBRIDGE, NA, United Kingdom; ^2^ University of Cambridge, Cambridge, United Kingdom

## Abstract

**Background:**

There is an urgent need for population‐specific predictive risk models for dementia that address earlier stages of the disease continuum. Blood‐based biomarkers (BBMs) have demonstrated excellent discrimination in high pre‐test probability populations, such as memory clinics. However, their performance in general population settings, where pre‐test probabilities are lower, remains underexplored. BBMs have the potential to enable earlier, targeted interventions, improve access to clinical trials, and enhance diagnostic accuracy in primary care. This systematic review synthesizes and meta‐analyzes the evidence on the predictive performance of BBMs in longitudinal models, focusing on their utility in primary care contexts.

**Method:**

A systematic review and meta‐analysis were conducted on studies evaluating the longitudinal predictive performance of BBMs, including *p*‐tau181, *p*‐tau217, *p*‐tau231, Aβ, GFAP, and NfL. Eligible studies included pre‐dementia populations reporting predictive performance using the area under the receiver operating characteristic curve (AUROC). Sensitivity analyses explored the impact of study setting, BBM combinations, baseline characteristics, and outcome (disease conversion vs. cognitive decline).

**Result:**

Of 205 full‐text studies reviewed, 32 met inclusion criteria, comprising models predicting dementia conversion (*n* = 20) or cognitive decline (*n* = 12). Most participants were from memory clinics (*n* = 20), fewer from community (*n* = 9) or combined settings (*n* = 3). Model reference covariates included age, sex, APOEε4 genotype, education (*n* = 24 models). Overall predictive performance was excellent, with pooled AUCs of 0.89 [95% CI: 0.85–0.92] in memory clinic populations and 0.85 [95% CI: 0.80–0.89] in community populations. Models stratified by symptomatology showed higher AUCs for symptomatic vs asymptomatic individuals (AUC=0.89, [95% CI: 0.86–0.91]) and (AUC=0.79, [95% CI: 0.76–0.78]). *p*‐tau181 performed better in single‐marker models (AUC=0.97, [95% CI: 0.94–1.00]) than in combination (AUC=0.87, [95% CI: 0.83–0.92]). Predictive performance was particularly strong in conversion from mild cognitive impairment (MCI) to Alzheimer's dementia (*p*‐tau181, AUC=0.91, [95% CI: 0.90–0.93]).

**Conclusion:**

Longitudinal BBM models demonstrate robust predictive ability for incident dementia and cognitive decline, even in the context of high heterogeneity across primary care and community settings. Early phases of disease, offer opportunities for stratified prediction. However, large observational trials in primary care are required to validate these findings, refine and optimise models for real‐world deployment.